# Health Tract, No. 10

**Published:** 1865

**Authors:** 


### HEALTH TRACT, No. 10.

### RULES FOR WINTER.

NEVER go to bed with cold or damp feet.

In going into a colder air, keep the mouth resolutely closed, that by compelling the air to pass circuitously through the nose and head, it may become warmed before it reaches the lungs, and thus prevent those shocks and sudden chills which frequently end in pleurisy, pneumonia, and other serious forms of disease.

Never sleep with the head in the draft of an open door or window.

Let more cover be on the lower limbs than on the body. Have an extra covering within easy reach in case of a sudden and great change of weather during the night.

Never stand still a moment out of doors, especially at street-corners, after having walked even a short distance.

Never ride near the open window of a vehicle for a single half-minute, especially if it has been preceded by a walk; valuable lives have thus been lost, or good health permanently destroyed.

Never put on a new boot or shoe in beginning a journey.

Never wear India-rubber in cold, dry weather.

If compelled to face a bitter cold wind, throw a silk handkerchief over the face; its agency is wonderful in modifying the cold.

Those who are easily chilled on going out of doors, should have some cotton batten attached to the vest or other garment, so as to protect the space between the shoulder-blades behind, the lungs being attached to the body at that point; a little there is worth five times the amount over the chest in front.

Never sit for more than five minutes at a time with the back against the fire or stove.

Avoid sitting against cushions in the backs of pews in churches; if the uncovered board feels cold, sit erect without touching it.

Never begin a journey until breakfast has been eaten.

After speaking, singing, or preaching in a warm room in winter, do not leave it for at least ten minutes, and even then close the mouth, put on the gloves, wrap up the neck, and put on cloak or overcoat before passing out of the door; the neglect of these has laid many a good and useful man in a premature grave.

Never speak under a hoarseness, especially if it requires an effort, or gives a hurting or a painful feeling, for it often results in permanent loss of voice, a life-long invalidism.

